# A Pilot Study of a Mobile Intervention to Support Mental Health and Adherence Among Adolescents Living with HIV in Western Kenya

**DOI:** 10.1007/s10461-021-03376-9

**Published:** 2021-07-22

**Authors:** Ashley Chory, Grant Callen, Winstone Nyandiko, Tabitha Njoroge, Celestine Ashimosi, Josephine Aluoch, Michael Scanlon, Carole McAteer, Edith Apondi, Rachel Vreeman

**Affiliations:** 1 Department of Health Systems Design and Global Health, Arnhold Institute for Global Health, Icahn School of Medicine at Mount Sinai, 1216 Fifth Avenue, Fifth Floor, Room 556, New York, NY USA; 2 Indiana School of Medicine, Indianapolis, IN USA; 3 Academic Model Providing Access to Healthcare (AMPATH), Eldoret, Kenya; 4 Moi Teaching and Referral Hospital, Eldoret, Kenya; 5 Department of Child Health and Paediatrics, School of Medicine, College of Health Sciences, Moi University, Eldoret, Kenya

**Keywords:** HIV, Adolescents, mHealth, WhatsApp, Kenya

## Abstract

Mobile technologies represent potentially novel and scalable intervention delivery platforms for adolescents living with HIV (ALWH) in low- and middle-income countries. We conducted a prospective, mixed methods pilot study to evaluate the acceptability and feasibility of the WhatsApp^®^ platform to deliver individual counseling services and facilitate peer support for ALWH in western Kenya. Thirty ALWH (17 female, mean age 15.4) on ART, engaged in HIV care and aware of their status, were enrolled. After 6 months, participants described their experiences with the intervention. Treatment adherence, stigma, and mental and behavioral health were assessed prospectively. Participants reported overall positive experiences and indicated that the platform encouraged peer network development. They endorsed potential benefits for treatment adherence, stigma reduction, and mental and behavioral health. All participants supported intervention expansion. In western Kenya, WhatsApp^®^ was an acceptable and feasible platform for mobile counseling and peer support for ALWH.

## Introduction

The advent of combined antiretroviral therapy (ART) transformed HIV from a terminal illness to a manageable chronic disease, yet HIV/AIDS remains a leading cause of death for adolescents aged 10–19 years globally, especially in sub-Saharan Africa (SSA) [1, 2]. Adolescents living with HIV (ALWH) face myriad inter-related clinical, social, and behavioral challenges to HIV treatment. ALWH often have high rates of loss to follow-up in clinical programs and low rates of adherence to treatment compared to adults and younger children [3–14]. There are few adolescent-specific clinical programs for HIV care, particularly in low- and-middle-income countries (LMIC), despite ALWH facing unique challenges—including transitions in care from pediatric to adult settings and increasing autonomy and control over their own health and behavior. For perinatally-infected ALWH, their HIV care includes disclosure of HIV status and the challenges that come with learning about their diagnosis and managing their treatment [15, 16]. HIV-related stigma and discrimination may negatively impact the development of peer networks and social support, which in turn affects all aspects of HIV care and treatment [17, 18], including both physical and mental health outcomes. ALWH experience higher rates of behavioral and mental health disorders and worse mental health outcomes compared to non-infected peers [19–22]. These challenges contribute to ALWH having higher rates of morbidity and mortality compared to other age groups [2], including high rates of viremia [23, 24] and drug resistance. Interventions targeting ALWH and their unique treatment and non-treatment related social and behavioral needs are essential to improving outcomes among this vulnerable population and addressing the inequitable treatment gap that this population faces.

Mobile-based technologies represent a potentially novel and scalable platform for delivering interventions targeting ALWH in LMIC. Mobile phone ownership in SSA has rapidly increased over the past decade; greater than 80% of Kenyans own a cellphone, with 15% owning a smartphone [25, 26]. Mobile phones have been used successfully among adults in resource-limited settings, as well as with adolescents in resource-rich settings, for HIV education and adherence support through SMS reminders [27–32]. Mobile phones have also been used to provide “tele-therapy,” with studies demonstrating the efficacy of both voice-calling and text messaging for the delivery of mental health interventions [33–38]. A 2017 randomized control trial in the rural United States conducted with adults living with HIV and comorbid major depressive disorder demonstrated that interpersonal therapy conducted via voice-calling, at 1 h per week for 9 weeks, served as an effective therapeutic intervention, with acute clinical improvement and high acceptability by participants [39]. A systematic review of tele-mental health interventions in LMIC demonstrated improvement in patients’ depression and aggression through different therapeutic delivery modalities—including videoconferencing, SMS, and voice-calling [40]. This body of literature shows that tele-therapeutic interventions can be effective in improving behavioral and mental health symptoms and in increasing access to mental health services, especially in LMIC. Beyond tele-therapeutic interventions, mobile platforms have been utilized as an avenue to provide peer support for ALWH, which may further promote social and behavioral health as well as adherence to treatment [41].

In this pilot study, we explored the acceptability and feasibility of a mobile-based counseling and peer support intervention for ALWH in HIV care in western Kenya.

## Methods

### Study Design

We conducted a prospective, mixed method study to evaluate the feasibility and acceptability of a mobile-based mental health and peer support intervention using the WhatsApp^®^ platform with ALWH in western Kenya. Thirty ALWH aged 10–19 years who were on ART, engaged in HIV care, and aware of their HIV status, were identified by clinic staff and recruited by the study team at the AMPATH-Turbo comprehensive care clinic in western Kenya. AMPATH[42, 43], the Academic Model Providing Access to Healthcare, is a long-standing partnership between a consortium of North American and Kenyan academic medical centers in partnership with the Kenyan Ministry of Health that provides comprehensive care for over 160,000 people living with HIV across western Kenya [44].

Participants were provided with a smartphone with the WhatsApp^®^ application preinstalled, a SIM card, and phone credit (~ 7 USD per month). ALWH enrolled in the study were placed in one of two WhatsApp^®^ groups based on their age, either a group for 9–14-year-olds or a second group for 15–19 year-olds. Each group had 15 study participants at baseline. A trained pediatric HIV adherence and disclosure counselor facilitated the WhatsApp^®^ groups according to a structured curriculum to encourage positive support between members, to introduce weekly group discussion topics, and to answer participants’ questions. Weekly group discussion topics were informed by formative qualitative work with this cohort as well as a multimedia curriculum developed previously by this research team that has modules on stress management, drug and alcohol abuse prevention, intimate relationships, and issues related to HIV adherence, disclosure, and stigma [45]. Participants were encouraged to use the WhatsApp^®^ groups to talk informally with other peers in the group; these conversations were monitored by the study counselor. In addition, the counselor contacted individual participants via direct WhatsApp^®^ messaging every other week throughout the duration of the study period to provide the opportunity for overall check-ins. Participants could contact the study counselor individually on an unscheduled, as-needed basis in the same manner. Group members participated in the mobile mental health support intervention for 6 months.

### Data Collection

During the 6-month intervention, we assessed participants’ adherence to HIV treatment, experiences with stigma, mental and behavioral health, and clinical status at baseline, 3 months, and 6 months. In addition, 25 participants participated in a pre-intervention interview to assess their familiarity and experience with mobile devices and WhatsApp^®^, mental and behavioral health knowledge, and perspectives on the HIV-related topics that they would find useful to be discussed in the WhatsApp^®^ groups. These data were used to inform the development of the WhatsApp^®^ platform-based intervention. Finally, we conducted post-intervention interviews with 15 participants to examine participants’ experiences during the intervention, including the acceptability and feasibility of delivering mental and behavioral health counseling and peer support through the WhatsApp^®^ platform.

### Measures

Acceptability and feasibility of the intervention was assessed via pre- and post-intervention interviews conducted by the study counselor in a private study room. Interviews were conducted in English or Kiswahili, depending on the preference of the participant and were audio-recorded and transcribed by the research team for analysis. Interviews were guided by a semi-structured interview guide that included topics on the feasibility and acceptability of the intervention components (e.g., clinical, social and behavioral topics discussed), format (e.g., how and when topics were discussed), and platform (e.g., mobile-based, WhatsApp^®^).

Adherence to HIV treatment was assessed using the Comprehensive ART Measure for Pediatrics (CAMP) Adherence Questionnaire, a short-form questionnaire that was developed and validated by this research team in western Kenya [46, 47]. The 10-item questionnaire includes questions on missed and late doses, as well as individual, household, and clinic-level barriers to adherence. In addition, all participants were provided with electronic dose monitors for continuous adherence monitoring using Medical Event Monitoring System (MEMS™, Westrock Belgium SPRL Rue Des Cyclistes Frontiere 24 Vise, 4600 Belgium), an electronic bottle cap that records the date and time of each bottle opening [48, 49]. MEMS have previously been shown to be a reliable measure of ART adherence that correlates with viral outcomes [48, 49]. HIV stigma was measured by the Stigma in AIDS Family Inventory (SAFI) questionnaire, which was also developed and validated in this setting among children and ALWH and their families [50]. The SAFI questionnaire assesses levels of perceived, enacted, and internalized stigma [50, 51].

Participants’ mental and behavioral health were measured using three widely used and validated instruments: the Patient Health Questionnaire-9 (PHQ-9), the Hopkins Symptom Checklist for Depression, and the Strengths and Difficulties Questionnaire (SDQ). The PHQ-9 depression tool is a 9-item questionnaire to screen for depression as described in the Diagnostic and Statistical Manual (DSM) of Mental Health Disorders IV [52–54]. Based on their responses, the PHQ-9 categorizes respondents into risk categories of “no depression,” “mild,” “moderate,” “moderate to severe,” and “severe,” with clinically significant depression indicated by a score beyond the moderate level [52–54]. The Hopkins Symptom Checklist for Depression consists of 25 questions, scaled between 0 and 4 points each, and assesses symptoms of depression [55]. This tool has been validated for use in East Africa among adult populations living with HIV, but not in children and adolescents. The SDQ assesses social and behavioral health by identifying respondents’ strengths and difficulties related to five different domains: conduct, peer problems, emotional symptoms, hyperactivity/inattention, and prosocial behavior [56]. Scores range from 0 to 40, with higher scores indicating more significant emotional and behavioral difficulties [56]. A threshold of 17 is used to determine an “abnormal” cut-off score and is correlated with social and behavioral difficulties [56]. The CAMP, SAFI, PHQ-9, SDQ, and Hopkins Symptoms Checklist were administered by the study counselor at baseline, 3, and 6 months in person at a participants’ regularly scheduled clinic visit. Answers were recorded on a paper form and entered into a REDCap database [57].

### Data Analysis

Acceptability and feasibility of the intervention was assessed via pre- and post-intervention interviews. Thematic analysis of the interviews was led by two researchers (TN and CA) who identified preliminary codes, which were entered into an Excel document and compared for consistency. Preliminary codes were further refined through review of the coding structure and analysis of the transcripts by two additional researchers (JA and MLS).

Descriptive analyses were conducted to assess clinical status, adherence to HIV treatment, stigma, and mental and behavioral health status of participants. Standardized tools such as the PHQ-9, SDQ, and Hopkins Symptoms Checklist were scored according to published guidelines. The SAFI stigma and CAMP adherence questionnaires have no standardized indices, so we analyzed responses to individual questionnaire items. We calculated adherence by MEMS in a few different ways: (a) a summation of the number of doses taken by the participant over the number of prescribed doses each month throughout the follow up period for a total average rate of adherence during the study (b) a dichotomized adherent versus non-adherent approach whereby to be “adherent” meant you took the minimum prescribed number of doses on more than 90% of days during follow up, and (c) if a participant had any treatment interruptions, defined as not taking medication for a period of more than 48 h. Given the small sample size, multivariable regression analyses were not conducted.

## Results

### Participant Demographics

Twenty-nine out of the 30 participants initially recruited completed the study and were included for analysis (one participant did not complete the study due to barriers experienced at boarding school that made it difficult to fully participate in the intervention). The mean age of participants was 15.4 years and the majority (56.7%, N = 17) were female.

### Acceptability and Feasibility: Pre-intervention Interview and Intervention Development

Twenty-five participants completed a pre-intervention interview. No participants reported owning a personal computer, and only one participant had previously owned a phone. The majority (77%) of participants had never had access to the internet or used any social media platforms to communicate with friends. Most participants (60%) reported some knowledge of mental health, but none reported a personal experience with mental health or utilizing mental health services. Participants mostly described mental illness as characterized by words like “madness,” “crazy” or “sickness,” and as being associated with major life disturbances or challenges. Participants associated the term depression specifically with suicidal ideation and self-destructive behaviors, anger, fighting, stress and general unhappiness. Participants were more familiar with the term anxiety, which was characterized as feelings of restlessness and fear. A minority (20%) of participants reported personally knowing someone with depression or anxiety.

### Acceptability and Feasibility: Post-intervention Interview Experiences in WhatsApp Group

Fifteen participants were randomly selected to complete a post-intervention interview, with roughly equal numbers from the younger and older WhatsApp^®^ groups. All participants reported positive experiences in the WhatsApp^®^ chats and reported that these groups created a feeling of community and peer support among ALWH that, for many participants, had not previously been available (Illustrative quotations of themes are provided in Table 1). Several participants reported that the paucity of previous peer support was secondary to a lack of interaction with other ALWH. Connecting ALWH to one another through these groups facilitated a novel platform to discuss issues that they had not been able to discuss before and provided a new sense of community.

“I was happy to be in the group, I learnt that I am not the only one who is positive because I used to be stressed a lot before I knew that we were many.”

“… sometimes someone would share something that I had already gone through and it would really help me know am not alone.”

**Table 1:** Benefits and challenges to using WhatsApp^®^ among ALWH

Theme	Illustrative quotes
Facilitation of relationships and communication	‘The group was good because it helped us have friends because I used to think I was alone, when I came to the clinic I did not have friends, but from that group, I got friends where we would talk and encourage each other, and in the WhatsApp, everybody was allowed to give their views’ ‘I was happy to be in the group, I learnt that I am not the only one who is positive because I used to be stressed a lot before I knew that we were many’
Access to a counselor	‘We talked about health issues, and hygiene and also asking about viral load’ ‘I was very stressed and bored with lif,e and I needed to talk to someone privately not in the group and it helped me feel good’
Medication adherence	‘It helped me by reminding me to take medicine… My friends would call to remind me to take medicine’ ‘The group was very educational, I liked it most that I have learnt about the right way to take the medicine’
Barriers to involvement	‘Sometimes, I would have a lot of work to do at home and homework and by the time I finished, it was already past 10 pm which would make me miss participating in the group’ ‘Most of the time, my phone would refuse to function, and this was the major challenge because I would go offline until it is serviced. Sometimes, my phone would spoil and sometimes not have power. This would make me be left behind and catching up on where I had left was a challenge’ ‘My parents used to keep the phone, and I would use it over the weekends’
Chat dynamics	‘It was good to put them [boys and girls] together so that we chat together and if there was something, they needed to discuss separately they can send in a private message’ ‘[I preferred] The group because, when you are two you don’t get a lot of ideas but in the group most people would come with different ideas which would be helpful’
Integrating WhatsApp counseling into routine care	‘Yes [you should expand the platform] because, patients in every clinic will learn that they are not alone and [participation in the group] would help in suppressing the virus’ ‘There are so many things that people don’t know and [Participation in the group] will help them learn’

All participants were contacted by the study counselor on a twice monthly basis. The majority (N = 23, 82%) of participants initiated at least one private communication with the counselor on WhatsApp^®^ outside of the group chats and reported such interactions to be helpful for the discussion of sensitive topics. Such discussions frequently included questions around medication adherence and general health and hygiene. Other times these discussions were more personal:

“I remember I had asked why I was the only one infected in our family—why did it choose only me?”

While one participant suggested the addition of a female-only group chat, the majority of participants liked mixed-sex groups and preferred group chats over individual chats. Participants also liked the use of nicknames to maintain confidentiality:

“Even if someone finds your phone they may not access it because if they see a different name they will think the phone is not yours.”

Participants identified household and school responsibilities as barriers to active participation, as well as logistical issues including power outages, insufficient phone credit, internet problems, and learning to use the mobile device. Caregivers were also mentioned by several participants as potential barriers to their participation in the intervention, with some caregivers not understanding why the adolescent was using the phone. Participants liked discussion topics that centered on hope, stress, stigma, and myths and misconception about mental health and HIV, as well as more clinical topics such as adherence to treatment.

All participants supported the potential expansion of the mobile-based counseling and peer support program to other clinics and identified several areas for improvement to support scaling up the program. Participants recommended utilizing school holiday breaks to identify and schedule in-person sessions with ALWH and counselors to see if they would be interested in participating. There were also several additional topics that participants proposed for further discussion in the WhatsApp^®^ chats, including sexual and non-sexual relationships with peers, managing conflict in their lives, and disclosure and confidentiality issues unique to ALWH.

### Mental and Behavioral Health, Stigma, and Adherence Outcomes

The results of the PHQ9, SDQ, and Hopkins questionnaires are shown in Table 2. Very few (N = 2) participants showed symptoms of depression via the PHQ9 and Hopkins measures. There was no discernable change over time in respondents’ scores on these assessments. In the SDQ, however, while the majority of participants had normal scores, about 5 participants had borderline or abnormal scores at each time point. Interestingly, it seemed that one domain was responsible for the majority of borderline scores; the mean participant score in the peer problems domain was consistently abnormal across each time point, while scores in other domains remained below the abnormal threshold—suggesting that peer-related issues are important to social and behavioral difficulties in this population.

**Table 2:** Mental and behavioral health outcomes

N (%)	At initial evaluation (n = 30)	3-month follow-up (n = 29)	6-month follow-up (n = 29)
PHQ			
None	29 (96.7)	29 (100)	28 (96.6)
Mild	1 (3.3)	0 (0)	1 (3.5)
SDQ total difficulties	12.70 (3.51)	12.57 (2.39)	13.83 (2.61)
SDQ difficulties category			
Normal	25 (83.3)	25 (86.2)	23 (79.3)
Borderline	3 (10.0)	4 (13.8)	6 (20.7)
Abnormal	2 (6.7)	0 (0)	0 (0)
Hopkins anxiety			
Normal	28 (93.3)	29 (100)	29 (100)
Symptomatic	2 (6.7)	0 (0)	0 (0)
Hopkins depression			
Normal	29 (96.7)	29 (100)	28 (96.6)
Symptomatic	1 (3.3)	0 (0)	1 (3.5)
Hopkins total			
Normal	29 (96.7)	29 (100)	29 (100)
Symptomatic	1 (3.3)	0 (0)	0 (0)

The SAFI stigma questionnaire results (Table 3) show that the most prevalent types of stigma-related issues for this group of ALWH were (a) feeling that it was important to keep their diagnosis a secret from others (63–76%) and (b) people in the community thinking HIV was an immoral, dirty, or shameful disease (23–52%). Reports of stigmatizing beliefs increased significantly (p < 0.5) throughout the study period with the majority (N = 15, 51.8%) of participants reporting that community members held stigmatizing beliefs about HIV. Several participants (16.7–20.7%) reported that they had missed taking their medication because they did not want others to see. While a small number of participants (N = 3) reported that their HIV status led them to view the future more negatively, several participants indicated that the WhatsApp^®^ groups gave them a sense of hope.

“What I liked most was the encouragement, because I had lost hope completely, I used to be very sick, very often I had persistent headaches and I would ask myself why I was born in this world to suffer but the group taught me to believe in God, and also having hope about the future.”

**Table 3:** HIV stigma outcomes

N (%)	At initial evaluation (n = 30)	3-month follow-up (n = 29)	6-month follow-up (n = 29)
I have lost friends	1 (3.3)	0 (0)	1 (3.5)
Called names/bullied	3 (10.0)	0 (0)	2 (6.9)
Discrimination at home	4 (13.3)	0 (0)	2 (6.9)
Discrimination neighbor	1 (3.3)	0 (0)	0 (0)
Discrimination church	0 (0)	0 (0)	0 (0)
Discrimination clinic	0 (0)	0 (0)	0 (0)
Stigma at school	1 (3.3)	0 (0)	1 (3.5)
Discrimination other	0 (0)	0 (0)	1 (3.5)
Loss of financial	0 (0)	0 (0)	0 (0)
Loss of social	0 (0)	0 (0)	0 (0)
Feel stressed/anxious	3 (10.0)	0 (0)	6 (20.7)
Feel depressed/sad	3 (10.0)	0 (0)	7 (24.1)
Don’t play/go places	1 (3.3)	1 (3.5)	1 (3.5)
Important keep secret	19 (63.3)	16 (55.2)	22 (75.9)
CG keeps secret	17 (58.6)	16 (55.2)	21 (72.4)
Future changed negative	5 (16.7)	1 (3.6)	3 (10.3)
Missed meds so people don’t see	5 (16.7)	2 (6.9)	6 (20.7)
Community thinks HIV dirty/shameful/immoral disease			
No one thinks that	23 (76.7)	20 (69.0)	14 (48.3)
A few people	4 (13.3)	9 (31.0)	14 (48.3)
Most	3 (10.0)	0 (0)	1 (3.5)

Adherence outcomes are presented in Table 4. Almost all participants were responsible for taking their own medication. Four (13%) participants reported problems taking their medication on time at baseline; only 3 (10%) reported problems at 6 months. At initial evaluation, seven participants (23%) reported missing at least one dose in the past 30 days, compared to only one participants at the 6-month follow-up visit. Adherence by MEMS electronic dose monitoring was lower compared to the self-report, with a mean adherence rate (average number of doses taken over prescribed) of 78%, with 59% of participants defined as “adherent” by taking their prescribed number of doses on > 90% of days during the 6-month monitoring period. Most participants (62%) had at least one treatment interruption of > 48 h across the study period. Several participants (N = 6, 40%) reported that the WhatsApp^®^ intervention had positive impacts on their adherence to treatment. When probed further, nearly every participant reported improved adherence related to one component of the intervention.

“It helped me by reminding me to take medicine… My friends would call to remind me to take medicine.”

**Table 4:** Adherence outcomes

	Baseline (N = 30)	3-month (N = 29)	6-month (N = 29)
CAMP Adherence Questionnaire			
Responsible for his/her own medication taking	30 (100)	29 (100)	28 (96.6)
Reported problems taking medication on time	4 (13.3)	0 (0)	3 (10.3)
Reported missed doses in past 7 days			
0	25 (83.3)	28 (96.6)	26 (89.7)
1	4 (13.3)	1 (3.5)	0 (0)
2	1 (3.3)	0 (0)	2 (6.9)
3	0 (0)	0 (0)	1 (3.5)
Reported doses taken more than 1 h late in past 7 days			
0	22 (73.3)	26 (89.7)	27 (93.1)
1	5 (16.7)	2 (6.9)	0 (0)
2	2 (6.7)	1 (3.5)	0 (0)
3	1 (3.3)	0 (0)	1 (3.5)
6	0 (0)	0 (0)	1 (3.5)
Reported missed doses in past 30 days			
0	21 (75.0)	27 (100)	28 (96.6)
1	2 (7.1)	0 (0)	0 (0)
2	2 (7.1)	0 (0)	0 (0)
3	2 (7.1)	0 (0)	0 (0)
6	1 (3.6)	0 (0)	0 (0)
8	0 (0)	0 (0)	1 (3.5)
Number of people in household taking HIV medication (including respondent)			
1	9 (30.0)	9 (31.0)	7 (25.0)
2	14 (46.7)	15 (51.7)	14 (50.0)
3	7 (23.3)	5 (17.2)	7 (25.0)
MEMS electronic dose monitoring adherence			
MEMS % of doses taken, mean (mean, SD)	78.2 (24)		
MEMS adherent > 90%, frequency (n, %)	17 (59%)		
1 or more MEMS treatment interruptions > 48 h (n, %)	18 (62%)		

## Discussion

Although this was a small pilot study, the evaluations of mental health, stigma, and adherence point to particular challenges related to peer problems, perceptions of HIV-related stigma and substantial issues maintaining ART adherence. We found that a mobile-based counseling and peer support intervention using the WhatsApp^®^ platform was both acceptable and feasible among a group of ALWH enrolled in care in western Kenya. The majority of participants had not previously had access to a phone and internet, despite high rates of mobile phone ownership in Kenya, likely due to their younger age and poorer socioeconomic status. Still, despite limited experience with the technology, participants reported that the WhatsApp^®^ groups provided valuable information and that the counseling services focused on issues that were important to them. Notably, participants expressed that they did not feel these topics were easily discussed with others (e.g., caregivers and healthcare providers) and that the groups therefore created a highly desired platform for community and social support. Many individuals indicated not knowing anyone their age who was also living with HIV. The WhatsApp^®^ groups therefore created an online peer network where they could freely discuss issues unique to ALWH in a safe space.

HIV-related stigma has been shown to have a significant impact on social support, mental health difficulties, and ART adherence [58–60]. Perceived stigma, or the fear of stigma occurring, may be especially relevant to this population—and specifically the fear of social isolation through the loss of peer networks and other resources [17, 61, 62]. On our stigma questionnaire, a majority of participants felt it was important to keep their HIV diagnosis a secret from others. In interviews with participants, the WhatsApp^®^ platform was liked in part because of the anonymity it allowed in a group counseling setting, with participants able to engage peers and counselors while also maintaining their privacy. The mobile platform and use of pseudonyms allowed participants to speak freely and share experiences without fear of retribution, allowing participants to discuss difficult topics—including stigma, non-adherence, and mental health challenges—more openly than in other settings. In the context of these conversations, our stigma questionnaire showed a significant increase in the number of participants identifying stigmatizing beliefs amongst community members from baseline compared to the end of the study. We hypothesize that this may be due to group discussions about stigma that provided participants with the concepts and language to identify and articulate stigmatizing attitudes, beliefs, and experiences. Similarly, the SAFI stigma questionnaire demonstrated a small increase in participants reporting issues with depression and anxiety at the end of follow-up, which may also result from participants’ increased mental health literacy.

Simultaneously, we found low rates of mental and behavioral disorders using the PHQ-9, SDQ, and Hopkins Symptoms Checklist. There may be several reasons for the discrepancy between outcomes across tools, including that few tools for mental health evaluation have been validated in this setting among ALWH. It is therefore possible that these tools may have underestimated the true burden of mental and behavioral challenges. Despite the fact that the SAFI tool is not a primary mental health evaluation, is has been validated in this setting, and therefore may be a better screening of these outcomes. We also hypothesize that a strong connection between anti-HIV stigma and its perception, which would be heightened in the context of a stigma evaluation, may lead participants to more readily report these feelings and symptoms. While there are few data on mental and behavioral health disorders among ALWH in Kenya and other LMIC [63, 64], the literature shows that psychiatric disorders, general psychological distress, and behavioral problems are a leading cause of health-related disability among children and adolescents worldwide—affecting 10–20% of the population [65–67]. Moreover, evidence demonstrates that only 1% of schools in LMIC had mental health professionals on staff [68] while there is an estimated one psychiatrist for every four million children in LMIC [69]. The fact that almost none of our study participants had ever been asked about their mental health and could not identify locations to seek services is consistent with this discrepancy in service availability. This is concerning as participants identified a need for a place to ask questions, find solutions to problems, and foster feelings of support, particularly related to mental health.

Given the lack of mental health services available for ALWH and the stigma surrounding it, opportunities to extend mental health services through alternative modes of delivery—such as teleconsultations and tele-psychiatry—may offer particular benefits for adolescents in LMIC [69]. A recent systematic review of randomized control trials assessing phone-based and computerized interventions for people living with HIV concluded that the delivery of mental health and adherence support through these modalities was generally acceptable to patients and effective at improving outcomes [33]. However, comparable interventions to ours, which includes mental and behavioral health education and counseling within peer groups among ALWH in LMIC, have not been rigorously evaluated. Mental health of children and adolescents must become a global health priority and central to ongoing efforts to end the HIV epidemic. This study demonstrated that counseling for youth living with HIV via a WhatsApp^®^ platform was well liked by participants and feasible for implementation in a low resource setting.

Finally, we found that adherence to treatment was a challenge in this cohort. This is consistent with literature regarding ALWH that demonstrate poor rates of adherence to treatment [70–72]. While this pilot study did not aim to assess the impact of our intervention on medication-taking, many participants reported that the WhatsApp^®^ groups helped them improve their adherence by establishing a peer support network. We believe that the design of this intervention is unique compared to previous mhealth interventions for adherence support that primarily utilize direct messaging for medication reminders [73, 74]. Our multidimensional pilot intervention, which incorporates education, counseling, and peer support components, is designed to address the root causes of non-adherence, such as stigma, a lack of social support, and mental health challenges.

There were several logistical challenges related to scheduling times for group discussions that all members could attend and infrastructural challenges such as power outages. In addition, we found that some participants reported that their phone was either taken away or used by their caregiver during the study period. Participants in boarding school had an especially difficult time using their phones as instructed, which led to one participant dropping out before the study’s end. These experiences illustrate typical challenges of adolescents who may have limited autonomy in their home and school environments. Overcoming these challenges requires education and coordination with parents, caregivers, and institutions so that adolescents can fully participate in intervention activities.

There were several limitations to this study. First, as a pilot study, there were only 30 participants enrolled, and they were only followed for 6 months. While we collected comprehensive data on behavioral and mental health, stigma, and adherence, it was not the purpose of this pilot study to measure the intervention’s impact on these outcomes. More intensive and longitudinal data on behavioral and mental health, stigma, and adherence are needed to better understand the potential impact of mobile-based counseling and peer support interventions among ALWH in this setting. Second, there were several challenges related to the intervention itself. For example, few participants had their own mobile phones or experience with the WhatsApp^®^ platform, as previously described. Expanding access to this intervention on a programmatic level in western Kenya would be expensive, as it would require the program to support both mobile phone ownership and phone credit to deliver the intervention for many ALWH.

## Conclusion

We demonstrated that WhatsApp^®^ platform was an acceptable and feasible strategy for delivering mobile counseling and peer support for ALWH in LMIC. Interventions should allow for both group and one-on-one messaging with a counselor in order to facilitate a sense of community with the option for private interactions to discuss more sensitive topics. Further data on the effectiveness and impact of mobile-based counseling and peer support interventions on mental health outcomes and adherence among ALWH in this setting are needed.
